# Intraoperative modulation of arterial blood flow in a hybrid operating room: A report of three cases

**DOI:** 10.1002/ccr3.2355

**Published:** 2019-08-15

**Authors:** Yoshikatsu Saitoh, Yasuyuki Hara, Shigehito Miyagi, Chikashi Nakanishi, Wataru Nakanishi, Ryuichi Nishimura, Daijirou Akamatsu, Hitoshi Goto, Michiaki Unno, Takashi Kamei

**Affiliations:** ^1^ Department of Surgery, Graduate School of Medicine Tohoku University Sendai Japan

**Keywords:** arterial embolization, general surgery, hybrid operating room, intraoperative modulation

## Abstract

The preoperative modulation of arterial blood flow is widely performed to prevent massive intraoperative hemorrhage and unstable circulatory dynamics; however, this may cause complications. The intraoperative modulation of arterial blood flow can be performed with operation to reduce the physical and psychological stresses on the patients and improve intraoperative safety.

## 1 INTRODUCTION

A hybrid operating room (HOR) refers to an operating room that is fully equipped with medical imaging devices, such as high‐quality angiographic equipment. A HOR is widely used for cardiac, vascular, and neurosurgical procedures as it allows for precise intraoperative catheterization and navigation.^1^, ^2^, ^3^, ^4^, ^5^ The use of intraoperative interventional radiology (IVR) in the HOR has been reported in cases of severe trauma and nephrectomy^5^, ^6^; however, its use has rarely been reported in the field of gastrointestinal surgery. Moreover, the role of a HOR in gastrointestinal surgery is yet to be established.

The intraoperative modulation of arterial blood flow (eg, arterial embolization) is widely performed to prevent massive intraoperative hemorrhage and unstable circulatory dynamics during the resection of large hypervascular tumors or splenectomy.^3^, ^7^, ^8^, ^9^, ^10^ However, complications of embolization (such as high‐grade fever, postembolic pain, pleural effusion, or transient hypertension) may occasionally necessitate the postponement or cancelation of elective surgery.^3^, ^11^ In addition, procedures performed in conscious patients may induce procedure‐related anxiety or psychological stress prior to surgery.

In an attempt to resolve these problems, we simultaneously perform planned catheterization and elective surgery under general anesthesia in the HOR. The objective of this case study was to highlight the potential applications of the HOR in the field of gastrointestinal surgery with invasiveness and hemorrhage by illustrating three representative cases.

## 2 CASE HISTORY

**Figure 1:**
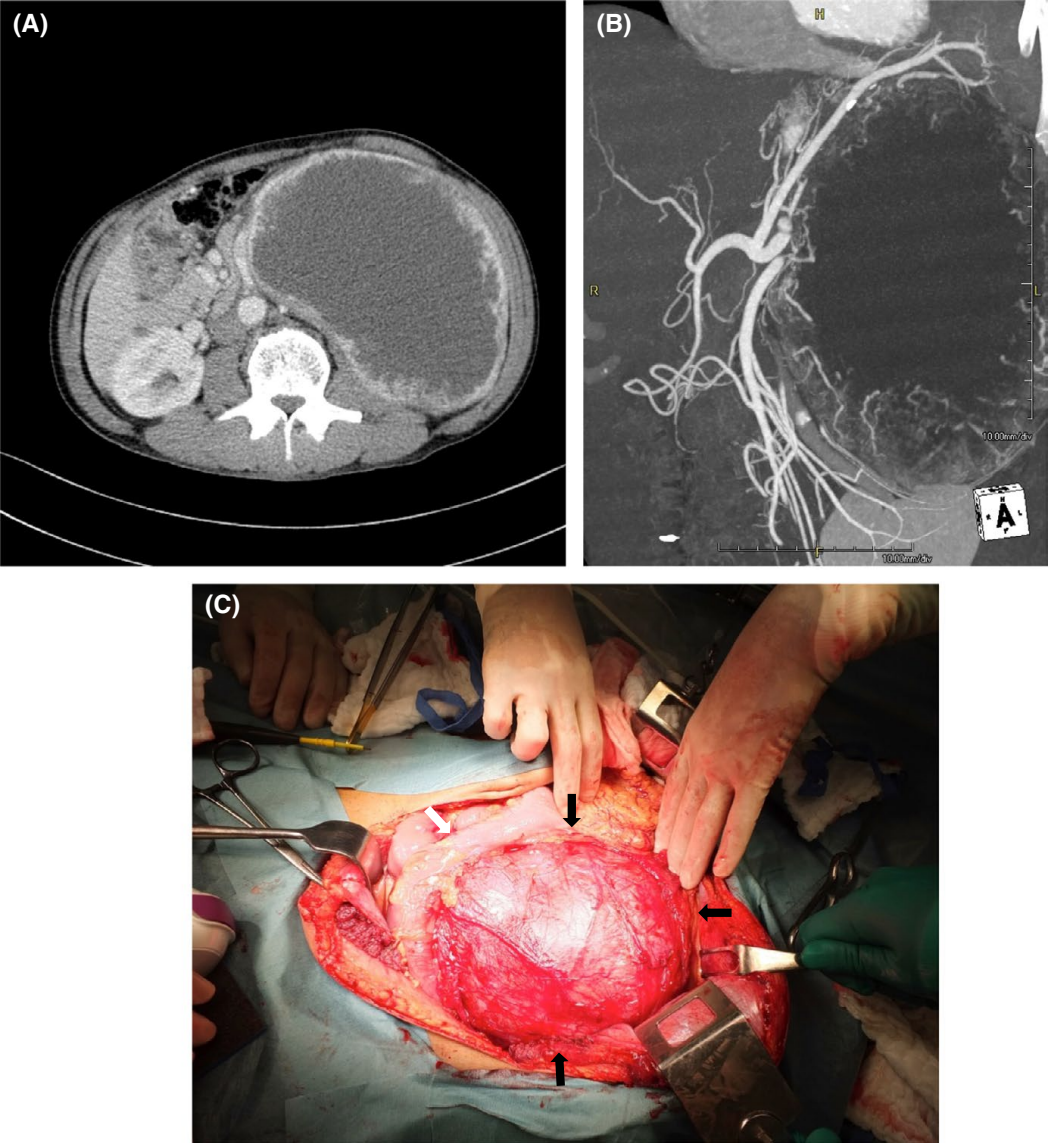
Case 1: A and B, preoperative abdominal computed tomography (CT) showing a giant., hypervascular left adrenal tumor measuring 21 × 19 × 12 cm. C, intraoperative photograph (laparotomy) showing a large pheochromocytoma (black arrows) at the caudal end of the transverse colon (white arrow)

The first patient was a 61‐year‐old man who initially presented to another hospital due to an abdominal tumor detected on medical examination. Computed tomography revealed a giant left adrenal tumor (size: 21 × 19 × 12 cm; Figure 1). A meta‐iodobenzylguanidine scan was positive, and the tumor was diagnosed as a pheochromocytoma.

The second patient was a 14‐year‐old boy. At the age of 9 months, following Kasai's operation for biliary atresia, he had undergone living donor liver transplant owing to liver cirrhosis. Following transplantation, he developed portal vein thrombosis and was administered anticoagulant therapy. However, portal hypertension was aggravated gradually, leading to gastric varices and splenomegaly (spleen size: 25 × 16 × 11 cm). Remarkable thrombocytopenia with gastrointestinal bleeding was also observed. Hassab's operation (devascularization of the proximal stomach and splenectomy) was scheduled at the age of 14 years.

The third patient was a 23‐year‐old man with a history of resection of retroperitoneal teratoma at the age of 29 days. Further, he had undergone left nephrectomy because of renal hypertension at the age of 80 days. Gastric varices, which were caused by the stenosis of the splenic vein due to previous operations, were detected at the age of 23 years, and Hassab's operation was scheduled.

### 2.1 Investigation and treatment

**Figure 2:**
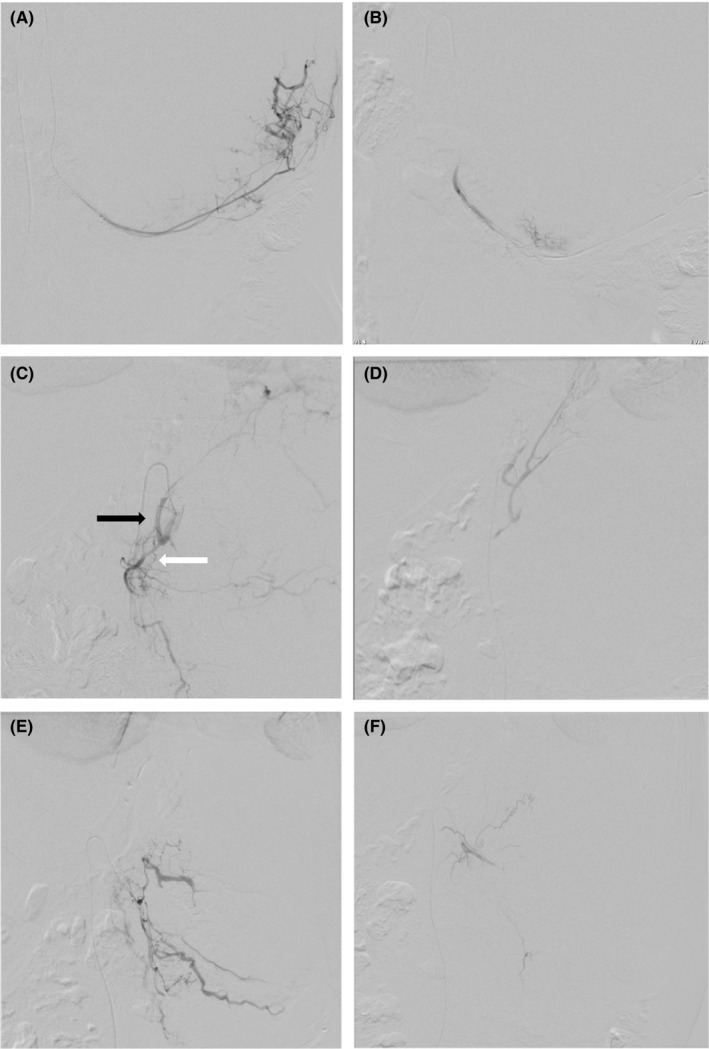
Case 1: Perioperative arterial embolization. A, angiogram of the left inferior suprarenal artery showing the artery feeding the tumor. B, postembolization angiogram of the left inferior suprarenal artery showing the disappearance of tumor vessels. C, angiogram showing the left inferior phrenic artery (black arrow) and the left superior suprarenal artery (white arrow). D, postembolization angiogram of both vessels showing the disappearance of tumor vessels. E, Angiogram of the left middle suprarenal artery. F, postembolization angiogram of the left middle suprarenal artery showing the absence of tumor blood flow

The first patient was perceived to be at high risk of massive intraoperative hemorrhage owing to the large tumor size and hypervascularity. In addition, there was a high risk of unstable circulatory dynamics due to changes in adrenal hormone levels. Consequently, we opted for perioperative arterial embolization in the HOR to ameliorate these risks. Under general anesthesia, the feeding arteries (left superior, middle, and inferior suprarenal arteries; left inferior phrenic artery) were embolized with a gelatin sponge through the right femoral artery (Figure 2). Circulatory dynamics remained stable during the embolization procedure, and laparotomy was performed. The tumor had not invaded the surrounding organs and was resected, with no massive intraoperative hemorrhage. Mild hypotension was observed following tumor resection, which was treated swiftly by the anesthesiologists. The estimated volume of intraoperative blood loss was 1281 mL.

**Figure 3:**
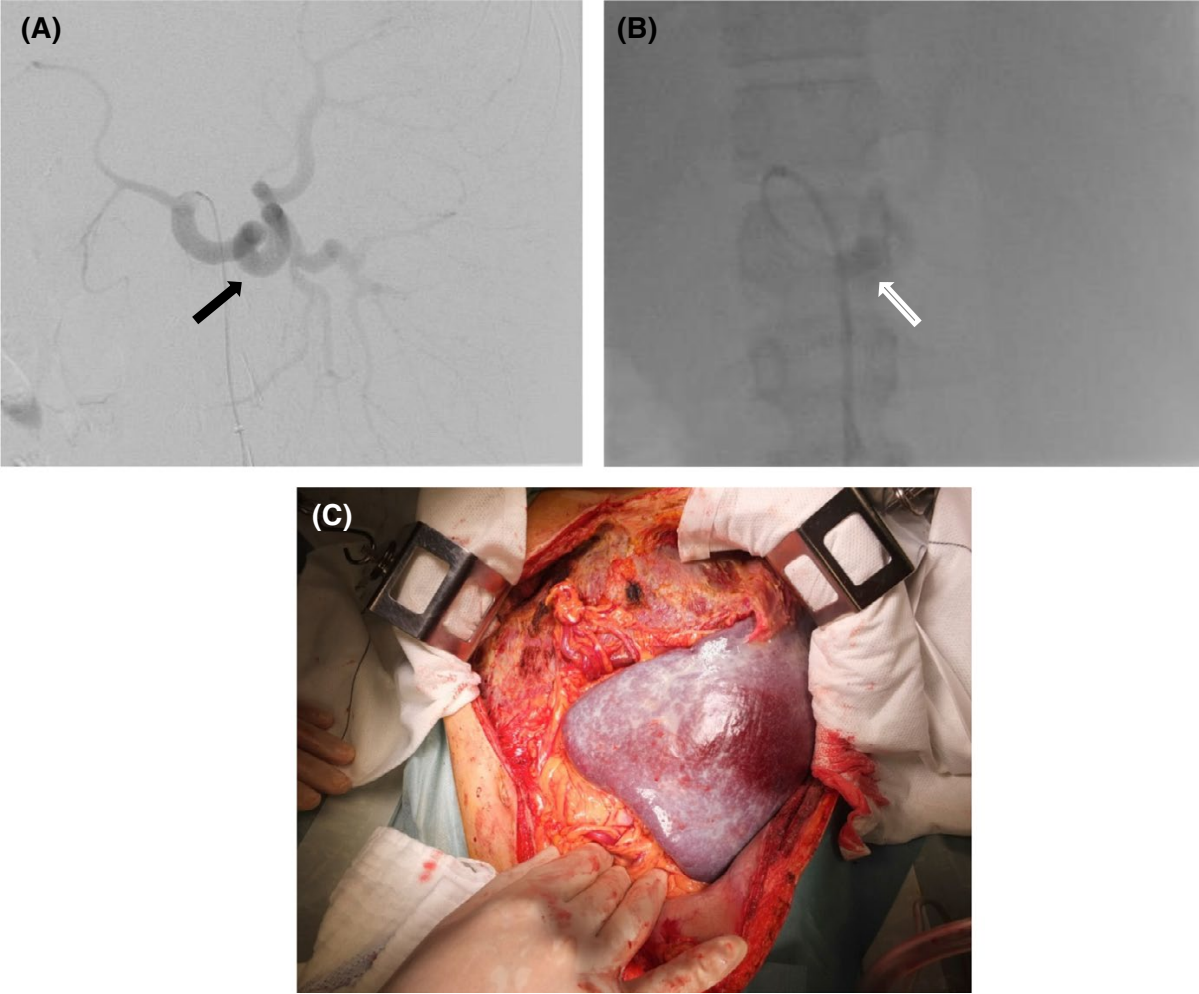
Case 2: Perioperative angiography. A, celiac axis arteriogram showing the splenic artery (black arrow). B, balloon occlusion (white arrow) has resulted in a near complete disappearance of blood flow to the spleen. C, intraoperative photograph at laparotomy showing a massive spleen (size: 25 × 16 × 11 cm)

In the second patient, the spleen was considerably enlarged, and the intraoperative control of the splenic artery was considered very difficult because of extensive adhesions caused by previous operations. There was also a high risk of massive intraoperative hemorrhage. Partial splenic embolization (PSE) was not performed before surgery. Because the second patient was a child, it was predicted that pain management after PSE would be difficult. Consequently, we opted for intraoperative occlusion of the splenic artery in the HOR. Under general anesthesia, balloon occlusion of the splenic artery was performed through the right femoral artery (Figure 3). Hassab's operation was performed immediately after catheterization. Intraoperative balloon occlusion assisted in identifying the splenic artery. The balloon was removed after the ligation and dissection of the splenic artery. The estimated intraoperative blood loss was 248 mL.

The third patient had a large spleen (16 × 14 × 9 cm), and the identification and control of the splenic artery was considered difficult because of extensive adhesions. Consequently, we opted to perform surgery in the HOR. Balloon occlusion of the splenic artery and Hassab's operation were performed in the same way as described in the previous patient. The estimated blood loss was 986 mL, and no blood transfusion was required.

### 2.2 Outcomes and follow‐up

The intraoperative blood loss in the three patients was 1281, 288, and 986 mL, respectively, and there were no perioperative complications. The postoperative course of all three patients was uneventful, and the patients were discharged 9, 16, and 18 days after surgery, respectively.

## 3 DISCUSSION

Operations performed in the HOR appear to effectively decrease intraoperative hemorrhage and help minimize postoperative complications.

The HOR is widely used at many institutions, and its benefits have been reported in many fields, such as cardiac, vascular, emergency, and neurosurgery.^1^, ^2^, ^3^, ^4^, ^5^ Furthermore, there are studies on the safety of intraoperative modulation in HOR in the fields of gynecology and gastrointestinal surgery.^12^, ^13^ In this study, IVR was performed by vascular surgeons or interventional radiologists. Close cooperation with IVR staff, anesthesiologists, radiological engineers, and nurses is vital to the success of operations performed in the HOR. Moreover, because the surgical tools and devices used in the HOR are identical to those used in a conventional operating room, open surgery might be performed in the HOR without additional stress.

The risk of intraoperative hemorrhage is an important consideration during operations for hypervascular tumors. High‐risk factors include very large tumor size, restricted range of tumor movement, and uncontrolled feeding arteries. In such cases, preoperative arterial embolization has been shown to be safe and effective for the decompression of tumors and reduction in intraoperative hemorrhage.^7^, ^8^, ^9^, ^10^, ^14^ In this study, the first patient had a very large and hypervascular pheochromocytoma, while the second and third patients had massive spleens. Consequently, controlling the splenic artery was considered difficult in all cases because of extensive adhesions caused by multiple surgeries. Therefore, arterial occlusion was recommended prior to resection and splenectomy.

Adrenal pheochromocytomas are catecholamine‐producing tumors that may cause perioperative hemodynamic changes, such as hypertension (resulting from anesthesia induction or adrenal gland manipulation) and prolonged hypotension (postresection^15^, ^16^). These hemodynamic changes have also been reported during IVR treatment alone.^16^, ^17^ However, if the patient is under general anesthesia, treatment for unstable circulatory dynamics can be initiated earlier and quicker. In this study, changes in blood pressure were not observed during arterial embolization, and only mild hypotension occurred after tumor resection. In the event of hemodynamic changes, anesthesiologists can treat these changes swiftly because of close monitoring under general anesthesia. Therefore, intraoperative procedures may improve the safety of arterial embolization and resection of pheochromocytomas.

In the cases of Hassab's operation, we opted for balloon occlusion to obtain arterial control. Balloon occlusion is a more convenient and faster approach than embolization. In addition, it facilitates the intraoperative identification of the splenic artery by palpation of the balloon. Therefore, balloon occlusion was considered useful for splenectomy in the setting of extensive postoperative adhesions.

Preoperative embolization may cause ischemia, necrosis, inflammation, and angiogenesis. Complications following embolization, such as high fever, postembolic pain, and pleural effusion, have also been reported.^3^, ^11^, ^14^ We presented PSE as an example of preoperative embolization. PSE is being performed since 1979; however, PSE is reported to be associated with complications. After PSE, fever of >38°C lasting for 3 days was observed in 53%, requirement for pain treatment in 24%, and pleural effusion in 6%.^18^

Complications may sometimes necessitate the postponement or cancelation of elective surgery. Moreover, procedures performed on patients in the conscious state tend to induce procedure‐related anxiety and psychological stress in them.^3^, ^19^ Intraoperative procedures may avert these complications and help reduce both physical and psychological stress for the patients.

This study has several limitations. First, this was an illustrative case study of a very small sample of patients operated at a single institution. Second, the patient selection criteria were heterogeneous. Thus, further research is required to evaluate the usefulness of this novel treatment strategy.

In conclusion, the intraoperative modulation of arterial blood flow in the HOR might be performed safely in the general surgical setting. This treatment strategy may reduce both physical and psychological stress in patients and improve the intraoperative safety.

## CONFLICT OF INTEREST

The authors have no conflict of interests.

## AUTHOR CONTRIBUTIONS

All authors designed the study. YH, SM, CN, WN, RN, DA, and HG: Were the principal surgeons. YS and YH: Reviewed the literature and ensured the quality of the manuscript. MU and YK: Revised the manuscript.
